# The role of the combination of echo-HRCT score as a tool to evaluate the presence of pulmonary hypertension in idiopathic pulmonary fibrosis

**DOI:** 10.1007/s11739-020-02539-1

**Published:** 2020-11-05

**Authors:** Rosa Metella Refini, Gloria Bettini, Esmeralda Kacerja, Paolo Cameli, Miriana d’Alessandro, Laura Bergantini, Ferdinando De Negri, Paola Rottoli, Piersante Sestini, Elena Bargagli, Maria Antonietta Mazzei

**Affiliations:** 1 Respiratory Diseases and Lung Transplantation, Department of Medical and Surgical Sciences and Neurosciences, Siena University Hospital, Viale Bracci 1, 53100 Siena, Italy; 2 Radiology Unit, Department, Azienda Ospedaliera Universitaria Pisana (AOUP), Pisa, Italy; 3 Department of Internal Medicine, University of Pisa, Pisa, Italy; 4 Unit of Diagnostic Imaging, Department of Medical, Surgical and Neuro Sciences and of Radiological Sciences, University of Siena, Azienda Ospedaliero-Universitaria Senese, Siena, Italy

**Keywords:** Pulmonary hypertension, Idiopathic pulmonary fibrosis, Algorithm, Right heart catheterization

## Abstract

Pulmonary hypertension (PH) is defined as an elevated mean pulmonary artery pressure at rest (mPAP ≥ 25 mmHg), evaluated by right heart catheterization (RHC). The aim of the present study was to evaluate HRCT findings in relation to transthoracic echocardiographic data to better characterize PH in IPF patients and to identify a non-invasive composite index with high predictive value for PH in these patients. 37 IPF patients were enrolled in this retrospective study. All patients underwent a complete assessment for PH, including transthoracic Doppler echocardiography, HRCT scan and right heart catheterization. Right heart catheterization was done in 19 patients (51.3%) as pre-lung transplant assessment and in 18 patients (48.6%) to confirm PH, suspected on the basis of echocardiography. Twenty out of 37 patients (54%) were confirmed to have PH by RHC. Multivariate regression showed that the combination of sPAP, PA area measured by HRCT and the ratio of the diameter of the segmental artery to that of the adjacent bronchus in the apicoposterior segment of the left upper lobe was strongly correlated with mPAP (*R*^2^ = 0.53; *p* = 0.0009). The ROC analysis showed that 931.6 was the ULN for PA area, with 86% sensitivity and 61% specificity (0.839 AUC); 20.34 was the ULN for the ratio of PA area to ascending aorta diameter, with 100% sensitivity and 50% specificity (0.804 AUC). The composite index proposed in the present study could help early detection of IPF patients suspected of PH requiring confirmation by RHC (if deemed clinically necessary).

**Electronic supplementary material:**

The online version of this article (10.1007/s11739-020-02539-1) contains supplementary material, which is available to authorized users.

## Introduction

Pulmonary hypertension (PH) is defined as an elevated mean pulmonary artery pressure at rest (mPAP ≥ 25 mmHg), evaluated by right heart catheterization (RHC). The latest clinical classification confirmed five major groups, based on pathological and hemodynamic characteristics [1, 2]. We focused on PH associated with idiopathic pulmonary fibrosis (IPF), which is the most common idiopathic interstitial pneumonia. Pulmonary hypertension associated with IPF is classified in group 3: PH due to lung disease and/or hypoxia [1, 3].

Idiopathic pulmonary fibrosis is a progressive and often fatal idiopathic interstitial pneumonia with a median survival of 3–5 years. It is limited to the lung and associated with a histological and/or radiological pattern of usual interstitial pneumonia [4]. The development of PH has a negative impact on quality of life of IPF patients and is associated with poor outcomes [5–7]. In recent years, it has been demonstrated that PH affects survival by increasing the risk of death [5–9].

The incidence and prevalence of PH in IPF are unclear and estimates vary widely due to underlying disease severity, patient population and diagnostic methods [9]. Currently, there is no specific therapy for PH associated with IPF. A recent clinical trial of a therapy produced negative results [10]; although two new molecules are now available for IPF treatment [pirfenidone and nintedanib], lung transplant is still the best choice in selected IPF patients to change the disease natural history and to improve survival [2, 4, 11].

The elective method of diagnosis of PH is RHC, an expensive invasive procedure with recognized complications, including hematoma (14%), pneumothorax (about 7%), arrhythmia (4%), episodes of hypotension [3%] and even death (0.055%) [12]; therefore, it requires an expert center and selected patients.^10^ Different studies have used a variety of other methods, contributing to the heterogeneity of the epidemiological data on PH in IPF [5, 9, 13].

Most data on the incidence of PH in IPF come from small cohorts, usually patients with advanced disease undergoing RHC as part of evaluation for lung transplant. These patients typically do not represent the general IPF population, usually being younger, more severe, and without significant medical comorbidities [6, 9].

Clinically, PH in IPF is difficult to recognize, and most of the symptoms are aspecific. Exertional dyspnea, as well as fatigue and palpitations are commonly found in both conditions [14]. Further appropriate tests are needed to diagnose PH when there is a strong clinical suspicion based on the severity of symptoms that exceed the expected, on the basis of lung function data or on the development of clear signs of right heart failure.

Echocardiography is a useful non-invasive technique for early investigation of suspected PH in IPF patients, although it cannot properly visualize all segments of the right heart and is operator dependent [2]. Non-invasive biomarkers, such B-type natriuretic peptide (BNP) and N-terminal pro-hormone BNP (NT-pro-BNP), have been proposed, but further work is needed before they can be applied in routine clinical practice [15–17].

High-resolution computed tomography (HRCT) of the chest is widely used as an integral part of diagnostic evaluation of patients with IPF. A number of features reflecting pulmonary hypertension can be measured by HRCT, but the literature is not unanimous about its accuracy in detecting PH [18–23]. HRCT and transthoracic echocardiographic measurements are both correlated with mPAP in lung diseases associated with PH and in idiopathic pulmonary arterial hypertension; a combination of the two provides complementary information that may improve the diagnostic power for PH [22, 23].

Since no non-invasive tool for diagnosis of PH in IPF patients has been identified and validated, RHC remains an elective technique.

The aim of the present study was to evaluate HRCT findings in relation to transthoracic echocardiographic data to better characterize PH in IPF patients and to identify a non-invasive composite index with high predictive value for PH in these patients.

## Materials and methods

Thirty-seven IPF patients, monitored and diagnosed from 2011 to 2019 at the Regional Referral Centre for Interstitial Lung Diseases at the University Hospital of Siena, were enrolled in this retrospective study. All patients underwent a complete assessment for PH, including transthoracic Doppler echocardiography, HRCT scan and right heart catheterization. Diagnostic imaging and radiological evaluation were performed at the Diagnostic Imaging Unit of the University of Siena, Italy. Inclusion criteria included diagnosis of IPF according to the latest evidence-based guidelines of the American Thoracic Society and European Respiratory Society/ALAT/JRS [4], pulmonary artery pressure measured by right heart catheterization performed for clinical purposes, chest HRCT and echocardiography within 1 month of RHC, and HRCT imaging performed at our institution. All patients were diagnosed during a multidisciplinary discussion by expert pulmonologists, radiologists, pathologists, rheumatologists, occupational doctors and internists. The population included IPF patients in waiting list for lung transplantation [24].

Patients with other types of interstitial lung diseases, such as ILD associated with connective tissue disease, drug-induced ILD, other types of idiopathic interstitial pneumonia, or patients with left heart disease, chronic pulmonary artery thromboembolism, or any cause of PH other than IPF were excluded from the study.

All patients underwent complete respiratory assessment with lung function tests, blood gas analysis and 6-min walking test performed according to international guidelines and good clinical practice. They gave their written informed consent to participation in the study, which was approved by our local ethics committee (CEAVSE 180,712; OSS_REOS 12,908).

### High-resolution computed tomography

HRCT was performed with a 64-row CT scanner (VCT, GE Healthcare, Milwaukee, WI, USA). All patients were imaged in prone position, with breath-holding at maximum lung capacity. HRCT examination was obtained in axial scanning mode for 12 patients and in spiral scanning mode for 25 patients. Acquisition parameters used in axial CT acquisitions were: 120 kV, 375 mA, 0.625 mm slice thickness (16i/rotation, 10 mm interval), 0.7 s rotation time, 50 cm sFOV. The images were reconstructed with the “bone plus” algorithm.

Acquisition parameters used in spiral CT acquisitions were: 140 kV; 250–330 mA, index noise 16, 3.75 mm slice thickness, 0.6 s tube rotation time, 0.937 beam pitch, 1.5 mm reconstruction interval. The images were reconstructed with a slice thickness of 1.25 mm using both the “bone plus” and “standard” algorithms.

**Fig. 1:**
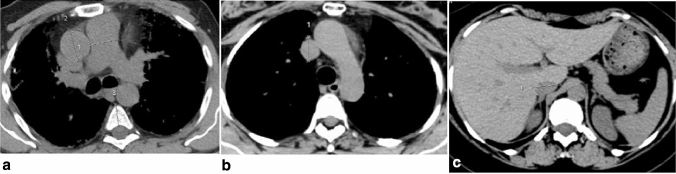
**a** The evaluated HRCT parameters: diameter of the *main pulmonary artery* (PA), before its bifurcation, and—on the same CT section—diameter of ascending aorta and the mid anteroposterior diameter of the thoracic vertebra. **b** The evaluated HRCT parameters: diameter of the superior caval vein. 1c: The evaluated HRCT parameters: diameter of the inferior caval vein

**Fig. 2:**
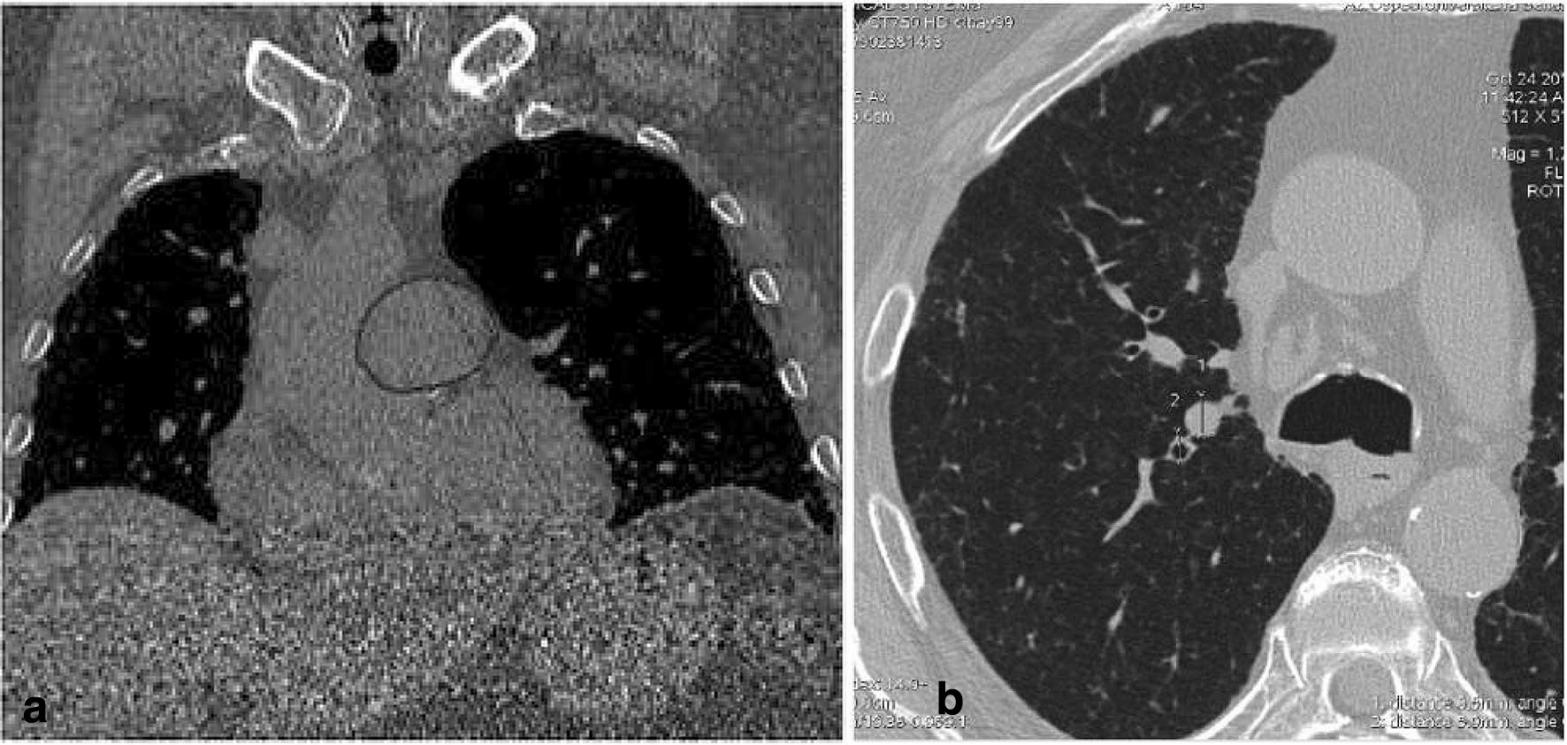
**a** The evaluated HRCT parameters: area of the *main pulmonary artery* (PA), before its bifurcation, on coronal reformation. **b** The widest short-axis diameters of the artery and bronchus of the apical segment of the right upper lobe

A radiology resident and an experienced thoracic radiologist, both blinded to the clinical and hemodynamic data, independently and retrospectively evaluated the HRCT scans. The interobserver agreement was obtained by applying a Kappa test. The Kappa unit ranged from 0 (chance agreement) to 1 (total agreement), in particular K values were deciphered in the following way: K < 0.20, poor agreement; K = 0.21–0.40, fair; K = 0.41–0.60, moderate; K = 0.61–0.80, good; K = 0.81–1.00, very good. Two authors (G.B, E.B.) examined the HRCT images independently and in a blinded fashion; radiological interpretation was made using the same criteria by visual quantitative analysis, as absent, mild (less 33% of lung involvement), moderate (between 33 and 66% of lung involvement) and severe (more than 66% of lung involvement). Divergent opinions were discussed and a consensus on final diagnosis was reached in all cases with the contribution of a third expert radiologist with 15 years of experience in diffuse lung pulmonary diseases (M.A.M). The HRCT parameters considered were: diameter and area of the main pulmonary artery (before the bifurcation) and, on the same CT section, diameter of the ascending aorta and mid anteroposterior diameter of the thoracic vertebra (a fixed structure that reflects the overall body size) (Figs. 1a, 2a); the widest short-axis diameters of the segmental arteries and bronchi of the following four lung segments: apical segment of the right upper lobe (Fig. 2b), apicoposterior segment of the left upper lobe, posterior basal segment of the right lower lobe, and posterior basal segment of the left lower lobe; diameters of the left ventricle and venae cavae (Fig. 1a–c). Evidence of emphysema, pericardial effusion, and hiatal hernia was also recorded.

### Echocardiography

During echocardiographic study, systolic pulmonary artery pressure sPAP was estimated by quantifying tricuspid regurgitant jet velocity (TRV) and inferior vena cava diameter/collapsibility index [25]. In all patients, the Doppler signal from tricuspid regurgitation was satisfactory. The tricuspid regurgitation pressure gradient (TRPG) was calculated according to the modified Bernoulli equation TRPG = 4 × (TRV)^2^ and sPAP was calculated from the equation sPAP = TRPG + estimated right atrial pressure.

### Right heart catheterization

Right heart catheterization was performed through the femoral or brachial vein and the following heart hemodynamic parameters were measured: systolic pulmonary artery pressure (sPAP), mean pulmonary artery pressure (mPAP), pulmonary capillary wedge pressure (PWP), pulmonary vascular resistance (PVR), cardiac index (CI) and cardiac output (CO). PH was defined as mPAP ≥ 25 mmHg and PWP < 15 mmHg evaluated by RHC.

### Lung function tests

The following lung function measurements were collected at the time of blood sampling (Table 3) and 6 months later: forced expiratory volume in the first second (FEV1), forced vital capacity (FVC), total lung capacity (TLC), residual volume (RV), diffusing capacity of the lung for carbon monoxide (DLCO) and carbon monoxide transfer coefficient (KCO) for alveolar volume. All parameters were expressed as percentages of predicted values. Measurements were performed according to ATS/ERS standards [26], using a Jaeger body plethysmograph with corrections for temperature and barometric pressure.

### Statistical analysis

A composite index was obtained combining HRCT and echocardiographic parameters and the formula obtained is reported in Fig. 1S.

Multivariate regression analysis was used to establish a composite index of mPAP from HRCT measurements combined with echocardiography parameters. The optimal cutoff or upper limit of normal (ULN) for a hypothetical quantitative predictor of PH was determined using receiver operating characteristic (ROC) analysis, where the ULN was deemed to be the value that yielded the best trade-off between sensitivity and specificity for each PH predictor. The relationship between HRCT parameters and mPAP was evaluated using linear regression analysis.

Statistical analysis was run using JMP v. 9.0 software (SAS Institute, Cary, NC).

## Results

The demographic data of our population monitored at Siena University Hospital from 2011 to 2019 is shown in Table 1. There were 30 male patients and 7 female patients with an average age of 63.7 years (range 51–78 years); 64.8% were smokers.

**Table 1:** Demographic data of IPF patients including age, gender and smoking habit

	IPF
N.	37
Age	63.7 ± 7 years
Gender	7 F (19 %), 30 M (81%)
Smokers	24 pts (64.8%)

Right heart catheterization was done in 19 patients (51.3%) as pre-lung-transplant assessment and in 18 patients (48.6%) to confirm PH, suspected on the basis of echocardiography (Table 2). Twenty out of 37 patients (54%) were confirmed to have PH by RHC (Table 3). No differences were observed regarding FEV1, FVC and DLco (*p* > 0,05) between groups.

**Table 2:** Classification of patients according to the reason for which they were submitted to RHC

	RHC done before lung transplantation	RHC done suspecting PH with echocardiography
Patients	19 pts (51.3%)	18 pts (48.6%)
IPF/PH	11 pts (55%)	9 pts (45%) PAPs DE 50 ± 14 mmHg
IPF/without IP	8 pts (47%)	9 pts (53%) PAPs DE 41 ± 10 mmHg

**Table 3:** Patients divided according to the presence of PH

	IPF/PH	IPF/noPH	
Patients	20 (54%)	17 (46%)	
Gender	18 M (90%) 2 F (10%)	12 M (70%) 5 F (30%)	
Age	64.7 ± 7.6 years	61.4 ± 5.8 years	
Smokers	13	11	
*Pulmonary function test parameters (median, IQR)*			
FEVI (%)	58 (43–83)	59 (42–77)	
FVC (%)	58 (39–74)	53 (42–74)	
DLCO (%)	21 (19–26)	31 (19–35)	
TLC (%)	56 (47–71)	60 (40–75)	

At the first reading, agreement, evaluated trough Cohen’s Kappa coefficient (k) between authors for different radiological elements, was excellent (*k* = 0.8–1) for the diameter of the segmental artery and good for diameter of the apicoposterior segment of the left upper lobe (*k* = 0.6–0.8).

**Fig.3:**
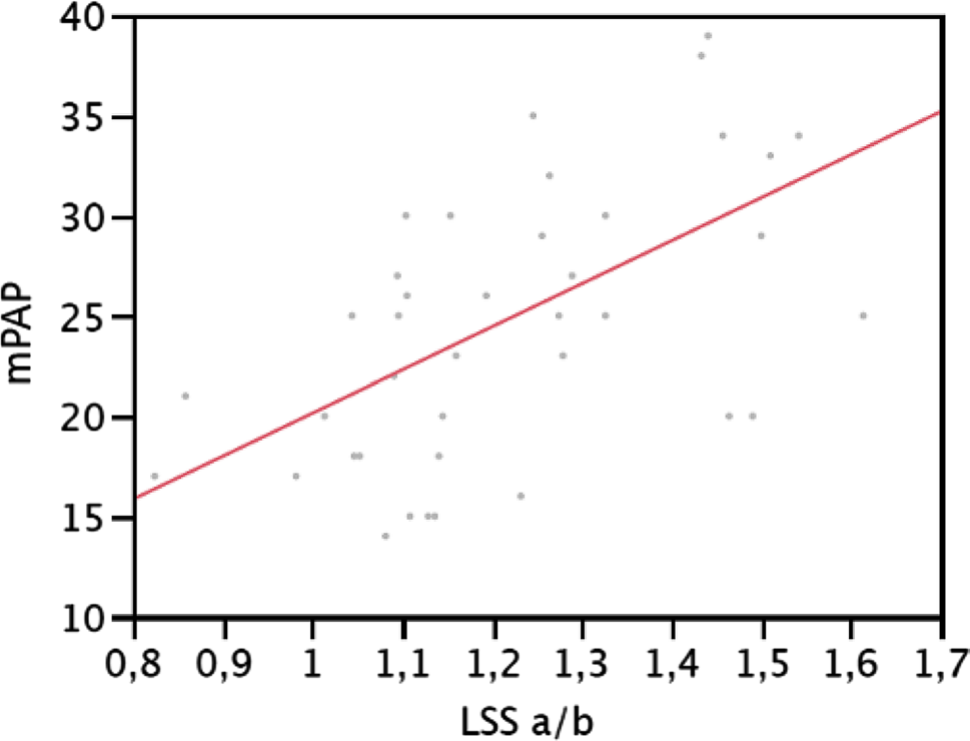
Linear regression between mean pulmonary artery pressure, measured at RHC (mPAP), and the ratio of the diameter of segmental artery to the adjacent bronchus in the left upper lobe (*R*^2^=0,349859; *p *= 0,0001)

The significant HRCT parameter related to mPAP was the ratio of the diameter of the segmental artery to that of the adjacent bronchus of the apicoposterior segment of the left upper lobe (*R*^2^ = 0.349859; *p* = 0.0001) (Fig. 3). Pulmonary artery cross-sectional area (*R*^2^ = 0.144669; *p* = 0.06) and the ratio of PA area to that of the ascending aorta (*R*^2^ = 0.131458; *p* = 0.0749) did not show a significant correlation with mPAP (Fig. 3), nor did we find any statistically significant correlation between mPAP and the other three artery:bronchus ratios.

Multivariate regression showed that the combination of sPAP, PA area measured by HRCT and the ratio of the diameter of the segmental artery to that of the adjacent bronchus in the apicoposterior segment of the left upper lobe were strongly correlated with mPAP (*R*^2^ = 0.53; *p* = 0.0009) (Fig. 1s). This composite index had 100% sensitivity and 53% specificity. Positive and negative predictive values were 71% and 100%, respectively. The contribution of other echocardiographic parameters (STRAIN and TTP) to the multivariate regression was not statistically significant.

The ROC analysis showed that 931.6 was the ULN for PA area, with 86% sensitivity and 61% specificity (0.839 AUC); and 20.34 was the ULN for the ratio of PA area to ascending aorta diameter, with 100% sensitivity and 50% specificity (0.804 AUC).

The combination of sPAP as measured by echocardiography, PA area measured by HRCT and the ratio of the diameter of the segmental artery to that of the adjacent bronchus in the apicoposterior segment of the left upper lobe was correlated with mPAP (*p* = 0.0009; *R*^2^ = 0.53).

## Discussion

Pulmonary hypertension is a risk factor for patients with IPF, being related to reduced survival; therefore it should be detected as soon as possible.

Chest HRCT is an accurate diagnostic instrument not only for the characterization of pulmonary fibrosis or other diffuse lung diseases, but also for simultaneously assessing the modifications in the vessel and heart structure induced by pulmonary hypertension [27–29]. A composite index obtained by combining HRCT and echocardiographic parameters has been found to be more accurate than the same parameters considered independently.

The challenge we addressed in this study was to find an alternative, non-invasive method of diagnosis of PH in IPF patients. Although RHC is the selective method, it is an invasive procedure, not without risks and unsuitable for follow-up in these cases. International guidelines, which recently highlighted the role of echocardiography in screening and follow-up of PH, may support this non-invasive diagnostic method in place of RHC. Since there is currently no specific therapy for PH associated with interstitial lung diseases, RHC can only be used when PH is suspected. A combination of chest HRCT and echocardiography could be useful to select IPF patients at high risk of PH to undergo RHC, apart from those assessed for lung transplant, for whom RHC is mandatory.

In our cohort, we demonstrated that a combination of HRCT and echocardiographic parameters could be used to spare RHC, reducing the risk and corresponding cost in 44.5% of patients (4/9) without PH, who did not require RHC for assessment for lung transplant.

In patients with PH who underwent RHC, despite an sPAP < 40 mmHg as measured by echocardiography (they were being assessed for the lung-transplant waiting list), the composite index indicated PH better than echocardiography, because of the high sensitivity, in agreement with RHC. The high negative predictive value of this index was not due to the low prevalence of PH in our study population, as 54% of patients had PH.

The literature shows that patients with PH may experience fluctuations in mPAP over periods of a few hours, suggesting the need to confirm the results with further data obtained by a group of tests (HRCT, DE and RHC) performed in a short period of time. In this perspective, HRCT is useful because it detects structural alterations due to PH. Devaraj et al. demonstrated that the ratio of the diameter of the main pulmonary artery to the diameter of the ascending aorta and the ratio of the cross-sectional area of the main pulmonary artery to the diameter of the ascending aorta were correlated with mPAP [22].

In our study, we did not find any statistically significant correlation between these parameters, including LFT parameters [7], although our population was more homogeneous than those of all previous studies. We only included patients diagnosed with idiopathic pulmonary fibrosis, while Devaraj's patients had a spectrum of diseases associated with PH (groups 1, 2, 3, 4 and 5 of the Venice classification system). Evaluating chest HRCT parameters in a consecutive cohort of acute hospitalized patients with different diagnoses, who underwent chest HRCT and RHC with a mean interval of 3 days, Chan et al. showed significant differences in mean PA/AA ratio between PH and non-PH patients. Most patients in the PH group showed elevated PWP due to congestive heart failure [30].

In a previous study, the ratio of the diameter of the main pulmonary artery to that of the ascending aorta in patients with PH associated with pulmonary fibrosis showed a weaker correlation with mPAP than it did in patients without pulmonary fibrosis [21]. Again, the population was heterogeneous, including different types of pulmonary fibrosis, not only IPF. Another limit of Devaraj’s last two studies was the range of the time interval between chest HRCT and RHC: up to 9 months [21, 22].

Using linear regression analysis, we found a strong correlation between mPAP and the ratio of the diameter of the segmental artery to that of the adjacent bronchus of the apicoposterior segment of the left upper lobe, but not between mPAP and the other three segmental artery: bronchus ratios. There is limited data in the literature on this issue. Devaraj showed that the segmental artery:bronchus ratio matched the severity of PH and was highly reproducible using a scoring system [22].

In most cases, diagnosis of PH is made in the late stages of IPF. Early recognition of PH in IPF patients is mandatory to allow them to participate in clinical trials. The high risk of mortality and poor prognosis associated with PH in IPF patients require special attention from clinicians. Also in patients evaluated for lung transplant, early detection of PH is important to correctly define transplant timing. Future therapeutic approaches are required to improve survival and the quality of life of these patients. The composite index proposed in the present study could help early detection of IPF patients suspected of PH requiring confirmation by RHC [if deemed clinically necessary]. Further studies are needed in a bigger prospective, cohort of IPF patients, also extending its application to other interstitial lung diseases associated with PH, to confirm its clinical utility.

## Electronic supplementary material

Below is the link to the electronic supplementary material.

Supplementary file1 (DOC 49 kb)

## Abbreviations

ILD: Interstitial lung disease
RHC: Right heart catheterization
PH: Pulmonary hypertension
IPF: Idiopathic pulmonary fibrosis
HRCT: High-resolution computed tomography

## References

[1] Galiè N, Humbert M, Vachiery J-L, Gibbs S, Lang I, Torbicki A (2015). ESC/ERS Guidelines for the diagnosis and treatment of pulmonary hypertension: The Joint Task Force for the Diagnosis and Treatment of Pulmonary Hypertension of the European Society of Cardiology (ESC) and the European Respiratory Society (ERS): Endorsed by: Association for European Paediatric and Congenital Cardiology (AEPC), International Society for Heart and Lung Transplantation (ISHLT). EurRespir J.

[2] Galiè N, Humbert M, Vachiery J-L, Gibbs S, Lang I, Torbicki A (2016). ESC/ERS Guidelines for the diagnosis and treatment of pulmonary hypertension: The Joint Task Force for the Diagnosis and Treatment of Pulmonary Hypertension of the European Society of Cardiology (ESC) and the European Respiratory Society (ERS): Endorsed by: Association for European Paediatric and Congenital Cardiology (AEPC), International Society for Heart and Lung Transplantation (ISHLT). Eur Heart J.

[3] Task Force for Diagnosis and Treatment of Pulmonary Hypertension of European Society of Cardiology (ESC), European Respiratory Society (ERS), International Society of Heart and Lung Transplantation (ISHLT), Galiè N, Hoeper MM, Humbert M, et al (2009) Guidelines for the diagnosis and treatment of pulmonary hypertension. Eur Respir J 34(6):1219–1263 10.1183/09031936.00139009 19749199

[4] Raghu G, Remy-Jardin M, Myers JL, Richeldi L, Ryerson CJ, Lederer DJ (2018). Diagnosis of Idiopathic Pulmonary Fibrosis. An Official ATS/ERS/JRS/ALAT Clinical Practice Guideline. Am J Respir Crit Care Med..

[5] Lettieri CJ, Nathan SD, Barnett SD, Ahmad S, Shorr AF (2006). Prevalence and outcomes of pulmonary arterial hypertension in advanced idiopathic pulmonary fibrosis. Chest.

[6] Patel NM, Lederer DJ, Borczuk AC, Kawut SM (2007). Pulmonary hypertension in idiopathic pulmonary fibrosis. Chest.

[7] Castria D, Refini RM, Bargagli E, Mezzasalma F, Pierli C, Rottoli P (2012). Pulmonary hypertension in idiopathic pulmonary fibrosis: prevalence and clinical progress. Int J ImmunopatholPharmacol.

[8] Kimura M, Taniguchi H, Kondoh Y, Kimura T, Kataoka K, Nishiyama O (2013). Pulmonary hypertension as a prognostic indicator at the initial evaluation in idiopathic pulmonary fibrosis. Respir Int Rev Thorac Dis.

[9] Rivera-Lebron BN, Forfia PR, Kreider M, Lee JC, Holmes JH, Kawut SM (2013). Echocardiographic and hemodynamic predictors of mortality in idiopathic pulmonary fibrosis. Chest.

[10] Behr J, Kolb M, Song JW, Luppi F, Schinzel B, Stowasser S (2019). Nintedanib and Sildenafil in Patients with Idiopathic Pulmonary Fibrosis and Right Heart Dysfunction. A Prespecified Subgroup Analysis of a Double-Blind Randomized Clinical Trial (INSTAGE). Am J Respir Crit Care Med..

[11] Vietri L, Cameli P, Perruzza M, Cekorja B, Bergantini L, d’Alessandro M, Refini RM, Pieroni M, Fossi A, Bennett D, Spalletti M, Mazzei MA, Sestini P, Rottoli P, Bargagli E (2020). Pirfenidone in idiopathic pulmonary fibrosis: real-life experience in the referral centre of Siena. Ther Adv Respir Dis.

[12] Hoeper MM, Lee SH, Voswinckel R, Palazzini M, Jais X, Marinelli A (2006). Complications of right heart catheterization procedures in patients with pulmonary hypertension in experienced centers. J Am Coll Cardiol.

[13] Andersen CU, Mellemkjær S, Nielsen-Kudsk JE, Bendstrup E, Hilberg O, Simonsen U (2013). Pulmonary hypertension in chronic obstructive and interstitial lung diseases. Int J Cardiol.

[14] Barst RJ, McGoon M, Torbicki A, Sitbon O, Krowka MJ, Olschewski H, et al (2004) Diagnosis and differential assessment of pulmonary arterial hypertension. J Am Coll Cardiol 43(12):40s–47s 10.1016/j.jacc.2004.02.032 15194177

[15] Leuchte HH, Baumgartner RA, Nounou ME, Vogeser M, Neurohr C, Trautnitz M (2006). Brain natriuretic peptide is a prognostic parameter in chronic lung disease. Am J Respir Crit Care Med.

[16] Leuchte HH, Neurohr C, Baumgartner R, Holzapfel M, Giehrl W, Vogeser M (2004). Brain natriuretic peptide and exercise capacity in lung fibrosis and pulmonary hypertension. Am J Respir Crit Care Med.

[17] Ruocco G, Cekorja B, Rottoli P, Refini RM, Pellegrini M, Di Tommaso C (2015). Role of BNP and echo measurement for pulmonary hypertension recognition in patients with interstitial lung disease: An algorithm application model. Respir Med.

[18] Ng CS, Wells AU, Padley SP (1999). A CT sign of chronic pulmonary arterial hypertension: the ratio of main pulmonary artery to aortic diameter. J Thorac Imaging.

[19] Pérez-Enguix D, Morales P, Tomás JM, Vera F, Lloret RM (2007). Computed tomographic screening of pulmonary arterial hypertension in candidates for lung transplantation. Transplant Proc.

[20] Zisman DA, Karlamangla AS, Ross DJ, Keane MP, Belperio JA, Saggar R (2007). High-resolution chest CT findings do not predict the presence of pulmonary hypertension in advanced idiopathic pulmonary fibrosis. Chest.

[21] Devaraj A, Wells AU, Meister MG, Corte TJ, Hansell DM (2008). The effect of diffuse pulmonary fibrosis on the reliability of CT signs of pulmonary hypertension. Radiology.

[22] Devaraj A, Wells AU, Meister MG, Corte TJ, Wort SJ, Hansell DM (2010). Detection of pulmonary hypertension with multidetector CT and echocardiography alone and in combination. Radiology.

[23] Condliffe R, Radon M, Hurdman J, Davies C, Hill C, Akil M (2011). CT pulmonary angiography combined with echocardiography in suspected systemic sclerosis-associated pulmonary arterial hypertension. RheumatolOxfEngl.

[24] Bargagli E (2018). PrasseA. Intern Emerg Med.

[25] Fields JM, Lee PA, Jenq KY, Mark DG, Panebianco NL, Dean AJ (2011). The interrater reliability of inferior vena cava ultrasound by bedside clinician sonographers in emergency department patients. AcadEmerg Med.

[26] Culver BH, Graham BL, Coates AL, Wanger J, Berry CE, Clarke PK, et al (2017) Recommendations for a Standardized Pulmonary Function Report. An Official American Thoracic Society Technical Statement. Am J Respir Crit Care Med 196(11):1463–1472 10.1164/rccm.201710-1981ST 29192835

[27] Valore della TC multistrato nella diagnosi di danno polmonare acuto/sindrome da distress respiratorio acuto 10.1701/1166.12889 23096732

[28] Abbritti M, Mazzei MA, Bargagli E, Refini RM, Penza F, Perari MG, Volterrani L, Rottoli P (2012). Utility of spiral CAT scan in the follow-up of patients with pulmonary Langerhans cell histiocytosis. Eur J Radiol.

[29] Sverzellati N, Odone A, Silva M, Polverosi R, Florio C, Cardinale L, Cortese G, Addonisio G, Zompatori M, Dalpiaz G, Piciucchi S, Larici AR (2018). Structured reporting for fibrosing lung disease: a model shared by radiologist and pulmonologist. La radiologia medica.

[30] Chan AL, Juarez MM, Shelton DK, MacDonald T, Li C-S, Lin T-C (2011). Novel computed tomographic chest metrics to detect pulmonary hypertension. BMC Med Imaging.

