# Supplementary figures and images for: The role of the combination of echo-HRCT score as a tool to evaluate the presence of pulmonary hypertension in idiopathic pulmonary fibrosis
Source: Intern Emerg Med. 2020 Nov 5;16(4):941–7. doi: 10.1007/s11739-020-02539-1 (PMC8195909; doi:10.1007/s11739-020-02539-1)

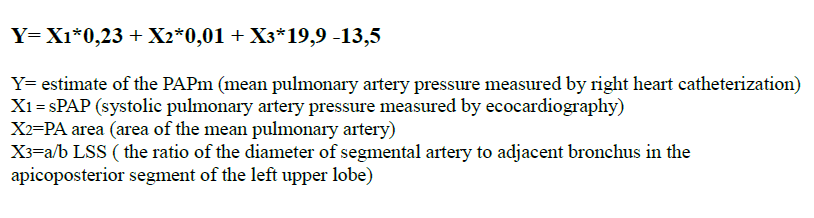


Fig.1s Multivariate regression analysis.

Supplement: Supplementary file 1 — Supplementary file1 (DOC 49 kb) [file 11739_2020_2539_MOESM1_ESM.doc]
